# Developing the content of two behavioural interventions:* Using theory-based interventions to promote GP management of upper respiratory tract infection without prescribing antibiotics #1*

**DOI:** 10.1186/1472-6963-8-11

**Published:** 2008-01-14

**Authors:** Susan Hrisos, Martin Eccles, Marie Johnston, Jill Francis, Eileen FS Kaner, Nick Steen, Jeremy Grimshaw

**Affiliations:** 1 Institute of Health and Society, Newcastle University, UK; 2 Department of Psychology, University of Aberdeen, UK; 3 Health Services Research Unit, University of Aberdeen, UK; 4 Clinical Epidemiology Program, Ottawa Health Research Institute and Department of Medicine, University of Ottawa, Ottawa, Canada

## Abstract

**Background:**

Evidence shows that antibiotics have limited effectiveness in the management of upper respiratory tract infection (URTI) yet GPs continue to prescribe antibiotics. Implementation research does not currently provide a strong evidence base to guide the choice of interventions to promote the uptake of such evidence-based practice by health professionals. While systematic reviews demonstrate that interventions to change clinical practice can be effective, heterogeneity between studies hinders generalisation to routine practice. Psychological models of behaviour change that have been used successfully to predict variation in behaviour in the general population can also predict the clinical behaviour of healthcare professionals. The purpose of  this study was to design two theoretically-based interventions to promote the management of upper respiratory tract infection (URTI) without prescribing antibiotics.

**Method:**

Interventions were developed using a systematic, empirically informed approach in which we: selected theoretical frameworks; identified modifiable behavioural antecedents that predicted GPs intended and actual management of URTI; mapped these target antecedents on to evidence-based behaviour change techniques; and operationalised intervention components in a format suitable for delivery by postal questionnaire.

**Results:**

We identified two psychological constructs that predicted GP management of URTI: "Self-efficacy," representing belief in one's capabilities, and "Anticipated consequences," representing beliefs about the consequences of one's actions. Behavioural techniques known to be effective in changing these beliefs were used in the design of two paper-based, interactive interventions. Intervention 1 targeted self-efficacy and required GPs to consider progressively more difficult situations in a "graded task" and to develop an "action plan" of what to do when next presented with one of these situations. Intervention 2 targeted anticipated consequences and required GPs to respond to a "persuasive communication" containing a series of pictures representing the consequences of managing URTI with and without antibiotics.

**Conclusion:**

It is feasible to systematically develop theoretically-based interventions to change professional practice. Two interventions were designed that differentially target generalisable constructs predictive of GP management of URTI. Our detailed and scientific rationale for the choice and design of our interventions will provide a basis for understanding any effects identified in their evaluation.

**Trial registration:**

Clinicaltrials.gov NCT00376142

## Background

Despite the considerable resources devoted to promoting the use of new evidence by clinicians, translating clinical and health services research findings into routine clinical practice is an unpredictable and often slow process. This phenomenon is apparent across different healthcare settings, specialties and countries, including the UK, [1-5] other parts of Europe [4] and the USA [5,6], with obvious implications for the quality of patient care.

Many systematic reviews of implementation interventions show that various interventions (e.g. reminder systems, interactive educational sessions) can be effective in changing health care professionals' clinical behaviour [7-11] but a consistent message is that these are effective only some and not all of the time. Why interventions have such variable success is difficult to establish as few of the studies reviewed to date provide an underlying theoretical basis to explain how or why an intervention might work [12]. Without such understanding of an intervention's "active ingredients" and what factors modify its effectiveness, there is little to guide the choice of intervention other than intuition or the knowledge that a similar intervention has been empirically successful in a previous study [9].

Interventions to implement evidence-based practice are often complex. The framework for the investigation of complex interventions suggested by the Medical Research council (MRC) [13] illustrates the current situation with implementation research (Table 1). To date most implementation research studies aiming to change clinicians' behaviour have involved trials at the exploratory or definitive randomised controlled trials (RCTs) stages of this framework, with few published studies providing evidence of preceding theoretical or modelling research. We aimed to address this gap in the current evidence-base through the development of a systematic intervention modelling process (IMP) for intervention development and evaluation that corresponds to each of the theoretical, modelling and experimental phases of the MRC Framework [14].

**Table 1:** Comparison of the stages in an evaluation of complex interventions to stages of drug evaluation.

**Evaluation of drugs**	Pre-clinical	Phase I	Phase II	Phase III	Phase IV
**Evaluation of implementation strategies**	Theory	Modelling	Exploratory trial	Definitive RCT	Long term implementation

Incorporating research findings into clinical practice almost invariably necessitates a change in clinical behaviour. Based on the idea that clinical behaviour is a form of human behaviour, we applied psychological models of behaviour change that have been used to predict variation in behaviour in the general population to the clinical behaviour of healthcare professionals. There is growing evidence to support the use of such theories in this way [15-17]. Psychological theory also underpins many behaviour change techniques for which there is evidence of effectiveness in changing the behaviour in other settings. Knowledge of the target behaviour or its cognitive antecedents is used to guide the selection of relevant interventions. For example, if individuals' beliefs about their capabilities relevant to a given task predict their behaviour, then their behaviour may be changed if they work through a series of tasks graded in order of increasing difficulty. This technique has been demonstrated to strengthen beliefs about capabilities.

This paper describes the process we used to design two theory-based interventions to promote the evidence-based management of upper respiratory tract infection, by GPs, without prescribing antibiotics. To enable experimental modelling and evaluation of the interventions prior to their use in a definitive RCT – which also forms part of the IMP -, the interventions were developed in the context of an "intervention modelling experiment" (IME) [16]. In an IME, key elements of an intervention are manipulated in a manner that simulates the "real world" as much as possible, but the measured outcome is an interim, or proxy, endpoint that represents the behaviour, rather than the actual behaviour itself. The evaluation of the interventions described here is reported in our partner paper [18].

## Methods

The process for the choice and development of the interventions was through a series of systematic steps, summarised in Table 2.

**Table 2:** Steps in developing a theory based behavioural intervention

1.	Specify target behaviour(s).
2.	Select theoretical framework (for empirical investigation at baseline and to assess process).
3.	Conduct a predictive study with a (preferably representative) sample drawn from the population of interest, to identify modifiable variables that predict the target behaviour(s) and their means/distributions. Based on the findings of this study, choose which variables to target. These variables are the proposed mediators of behaviour change.
4.	Map targeted variables onto behaviour change techniques and select techniques that (a) are likely to change the mediator variables and (b) it is feasible to operationalise.
5.	Choose appropriate method(s) of delivery of the techniques.
6.	Operationalise intervention components (techniques) in appropriate combination and order.

Note: As part of an iterative process, results from the implementation modelling experiment will provide information for feedback loops that address earlier points in this sequence. This feedback loop permits change, development or refinement of the intervention.

### Specification of the target behaviour/s

The consultation for upper respiratory tract infection (URTI) is one of the most frequent in general practice [19]. Research evidence has shown that antibiotics are of limited effectiveness in treating URTI [20-22]. However, GPs continue to manage patients with uncomplicated URTI by prescribing antibiotics [23,24]. In specifying our target behaviour, we used the "TACT" principle, a systematic way of defining behaviour in terms of its Target, Action, Context and Time [25]. For the behaviour, "managing patients presenting with uncomplicated URTI without prescribing antibiotics", the target is the patient, the action is managing without prescribing an antibiotic, the context is the clinical condition (uncomplicated URTI) and the time is during a primary care consultation.

### Selection of the theoretical framework

Our choice of theoretical framework was guided by the findings of a previous study by the authors which explored the utility of a range of psychological models in identifying provider-level factors predictive of clinical behaviour [26]. This study found that three theories included constructs that predicted GPs' prescribing behaviour for URTI: Theory of Planned Behaviour (TPB) [27], Social Cognitive Theory (SCT) [28,29] and Operant Learning Theory (OLT) [30]. These theories explain behaviour in terms of factors amenable to change (e.g. beliefs, perceived external constraints); and they include non-volitional components that acknowledge that individuals do not always have complete control over their actions. They have also been rigorously evaluated in other settings, providing a sound scientific basis for the development of interventions.

According to the TPB, specific behaviours can be predicted by the strength of an individual's intention to enact that behaviour. Intentions are thus the precursors of behaviour and the stronger the intention, the more likely it is that the behaviour will occur. Intention is, in turn, influenced by the individual's attitudes towards the behaviour; their perceptions of social pressure to perform the behaviour ("subjective norms"); and the extent to which they feel able to perform the behaviour ("perceived behavioural control"). SCT considers self-efficacy (confidence that one is able to perform the behaviour), outcome expectancy (an individual's estimate that a given behaviour will lead to certain outcomes), risk perception and individuals' goals in explaining behaviour, including proximal goals (such as intentions). OLT proposes that behaviours that have contingent consequences for the individual are more likely to be repeated when the individual's "anticipated consequences" of their behaviour are favourable, and will become less frequent if their anticipated consequences are less positive. OLT also proposes that behaviours performed frequently in the same situation are likely to become habitual (automatic) [30].

These theoretical frameworks allow the identification of potential causal pathways underlying behaviour change (i.e. evaluation of thought processes that explain behaviour change). Within any subsequent evaluation of the impact of the intervention being developed, the measurement of potential mediators of behaviour change targeted by an intervention allows an understanding of the causal mechanisms involved in the change. This is one part of a "process evaluation".

### Identification of constructs to target for change

In addition to guiding our choice of theoretical framework, we also used the findings of Eccles *et al.*[26], to identify which constructs to target with our interventions. In that study, a random sample of GPs from Scotland were surveyed about their views and experiences of managing patients with uncomplicated URTI. Theory-based cognitions were measured by a single postal questionnaire survey during a 12 month period. Two interim outcome measures of stated intention and behavioural simulation were collected at the same time as the predictor measures. GPs' simulated behaviour was elicited using five clinical scenarios describing patients presenting in primary care with symptoms of an URTI. GPs were asked to decide whether or not they would prescribe an antibiotic and decisions in favour of prescribing an antibiotic were summed to create a total score out of a possible maximum of five. Data on actual prescribing behaviour were also collected from routinely available prescribing data for the same 12 month period. Analyses explored the predictive value of theory based cognitions in explaining variance in the behavioural data (Table 3).

**Table 3:** Summary of the systematic selection of theoretical constructs to target in the development of the interventions^1^.

**Theoretical Construct**	**Intention**		**Simulated Behaviour**		**Behaviour**		
**TPB**	Predictor Y/N	r	Predictor Y/N	r	Predictor Y/N	r	**Mapped beliefs that discriminate between GPs who do and do not intend to manage URTI without antibiotics [17]**

Attitude direct*	**Y**	0.49	**Y**	0.32	N	0.07	
Attitude indirect*	**Y**	0.41	**Y**	0.21	N	0.02	
Intention	-	-	**Y**	0.44	**Y**	0.19*	
PBC direct	**Y**	-0.28	**Y**	-0.39	N	-0.04	
PBC indirect	**Y**	0.60	**Y**	0.49	N	0.17*	
Subjective norm	**N**	0.04	**N**	0.005	N	-0.10	
**SCT**							
**Risk perception	**Y**	0.54	**Y**	0.35	**Y**	0.17*	• Prescribing an antibiotic for these patients will reduce their risk of developing minor complications such as otitis media and sinusitis (BB) • Because I don't know the cause of these patients' sore throats, I will prescribe an antibiotic so that I don't miss something (CB) • In most cases, the patient will finish the course of antibiotics I prescribe(CB)
Outcome expectancy (2 items)	**Y**	0.41	**Y**	0.19	N	-0.05	
Outcome expectancy (7 items)	**Y**	0.21	**Y**	0.27	N	-0.03	
**Self-efficacy**	**Y**	0.56	**Y**	0.43	**Y**	0.14*	• If a patient asks for an antibiotic then I will prescribe one whether it is medically indicated or not (CB) • I am more inclined to prescribe an antibiotic for patients of a lower social class (CB) • Because I don't know the cause of these patients' sore throats, I will prescribe an antibiotic so that I don't miss something (CB) • In most cases, the patient will finish the course of antibiotics I prescribe (CB)
**OLT**							
**Anticipated consequences	**Y**	0.54	**Y**	0.35	**Y**	0.17*	• Prescribing an antibiotic for these patients will reduce their risk of developing minor complications such as otitis media and sinusitis (BB) • Because I don't know the cause of these patients' sore throats, I will prescribe an antibiotic so that I don't miss something (CB) • In most cases, the patient will finish the course of antibiotics I prescribe (CB)
**Evidence of habitual behaviour**	**Y**	0.64	**Y**	0.46	**Y**	0.23*	

1. Data from interim analysis of dataset [25]

* TPB attitudes and PBC constructs can be measured "indirectly" by asking individuals to report their specific beliefs or directly by asking individuals to report at a more general level

**The SCT risk perception questions were also used as a measure of OC anticipated consequences. CB = Control Belief; BB = Behavioural Belief

In considering the most important constructs to target in this modelling experiment we selected constructs that were significantly correlated with GPs' actual behaviour (rates of prescribing antibiotics). There were five candidate psychological constructs: Intention (TPB); risk perception and self-efficacy (SCT) and anticipated consequences and evidence of habitual behaviour (OLT) (Table 3). Scores on these constructs were also significantly correlated with behavioural simulation scores. As Intention was also to be a dependent variable in the modelling experiment it was not appropriate to directly target this construct. Habitual behaviour was also not selected as a target variable as it is not a causal determinant but rather an attribute of behaviour, and is modified indirectly by targeting other causal aspects of behaviour. The remaining three constructs: self-efficacy, risk perception and anticipated consequences were the theoretical constructs chosen as targets for our interventions.

### Mapping targeted constructs onto behaviour change techniques

In choosing the most appropriate behaviour change techniques for the target constructs, we first mapped the three target constructs onto the theoretical construct domains identified by Michie et al (2005) [31] (Table 4). We then used a recently developed tool which further maps these theoretical construct domains on to behaviour change techniques [32]. This tool documents expert consensus on the use of 35 behaviour change techniques as appropriate interventions to change each construct domain. The techniques are supported by evidence of their effectiveness [33].

**Table 4:** Mapping of target constructs to construct domain & behavioural change techniques

**Target Construct**	**Construct Domain**	**Behavioural Change Techniques**
**Self-Efficacy (SCT)**	Beliefs about one's capabilities	• Self-monitoring • **Graded Task** • Increasing skills • Coping skill • Rehearsal • Social pressure • Feedback • Self-talk • Motivational interviewing

^1^Risk perception ^1^Anticipated consequences	Beliefs about the consequences of one's action	• Self-monitoring • **Persuasive communication** • Information regarding behaviour outcome, connection between the two • Feedback

^1^Risk perception & Anticipated consequences are similar constructs and use a shared measure.

### Choose an appropriate method of delivery

A paper-based method of delivery of the intervention was chosen because, recognising the geographical spread of the sample, for a subsequent evaluation greater efficiency would be obtained if the experiment could be administered by post.

### Operationalising the intervention components

Different ways of operationalising the interventions as paper-based tasks were developed using an iterative process involving the study team members (MJ, JF, SH, EFK & ME). It was important to recognise that a paper-based format might be a relatively passive means of delivering the intervention components. Hence to limit this possibility, the interventions were operationalised to maximise the interactive nature of each intervention component.

## Results

Two interventions were developed, directed at changing different constructs. The first intervention targeted the theoretical construct of self-efficacy (from SCT). This construct mapped on to the theoretical construct domain, "beliefs about capabilities". The main behaviour change technique selected was "graded task" [29]. The aim of this intervention was to increase GPs' beliefs in their capabilities of managing URTI without prescribing antibiotics. The graded task technique does this by promoting incrementally greater levels of "mastery" by building on existing abilities, demonstrating success at each level. Two further behaviour change techniques, "rehearsal" and "action planning" were additional components of this intervention. The "rehearsal" technique used the generation of alternative strategies as a way of rehearsing alternative actions that could be applied to the clinical situation. The "action planning" technique involved asking the participants to develop a plan of actions they intended to take when confronted by a clinical situation in which a patient presented with an URTI. Interventions are named according to the principle behaviour technique used.

• ***Graded Task intervention ***(Additional file 1): Recipients were presented with five situations in which GPs would be required to manage a patient presenting with sore throat. The situations were derived from questionnaire items used in the predictive survey [17] and ranked in order of difficulty based on the responses to these questions by GPs. Starting with the easiest, respondents were asked to consider each of these situations in turn, and to indicate if they could confidently manage the patient without prescribing an antibiotic. The response format was "Yes," "Maybe" and "No". Thus the typical pattern of responses would be a series of successes ("yes") before a series of failures ("no") in response to more difficult situations. They were then asked to select the situation that they found the least difficult to achieve from those they had rated as "Maybe" or "No," and write the number of this situation in a box provided. If they had rated all of the situations listed as "Yes," they were asked to write down a related situation that they would find difficult to achieve. Focusing on their selected situation, participants were then instructed to a) generate possible alternative management strategies for that situation and then b) to develop a plan of what they would do to manage this situation in the future.

The second intervention targeted the theoretical constructs of anticipated consequences (from OLT) and risk perception (from SCT). These constructs both mapped on to the theoretical construct domain "beliefs about consequences". The behaviour change technique selected was "persuasive communication." The aim of this intervention was to encourage GPs to consider some potential consequences for themselves, their patients and society of managing URTI with and without prescribing antibiotics. This intervention also incorporated elements of the behaviour change technique, "provide information regarding behaviour, outcome and connection between the two" (Table 4).

• ***Persuasive Communication intervention ***(Additional file 2): This intervention presented GPs with two sequences of five pictures illustrating some possible consequences of managing URTIs with or without antibiotics. The consequence illustrated in each fictitious situation depicted was created to reflect the content of questionnaire items used by Eccles *et al.*[26] to ask about risk perception and anticipated consequences; and the discriminant beliefs identified by Walker *et al.*[17] as predictive of GPs who do and do not intend to manage URTI without antibiotics. The first row of pictures represents "Dr A", who manages URTI by prescribing antibiotics and the second row representing "Dr B", who manages URTI without prescribing antibiotics. To highlight the suggested consequences and to help recipients relate these possible consequences to each doctor's prescribing behaviour, questions were placed beneath each picture. Participants were not required to respond to these questions. However, to further enhance the interactive nature of this intervention GPs were asked to indicate on a bi-polar analogue scale a) the extent to which they try to be like Dr A or Dr B (i.e. their "intended" behaviour) and b) the extent to which they are actually like Dr A or Dr B (i.e. their "actual" behaviour).

## Discussion

A major problem with implementation research to date has been the limited understanding about what interventions contain and how they are meant to work. Contributing to this is the frequently scant, or absent, reporting of the process of intervention development. In addition, few studies provide a theoretical basis for the choice and design of interventions to change clinical practice. We have developed an intervention modelling process (IMP) that corresponds closely to the theoretical and early modelling phases of the MRC Framework [13] – explicit stages of development that are currently lacking in implementation research. The systematic approach we have used here in the development of the content of two theory-based behavioural interventions forms the initial part of the IMP.

The contents of the interventions were designed to differentially target specific "determinants of behaviour change" – theoretical constructs that were identified in a previous study as predictive of both the behaviour and the intention of GPs to manage URTI without prescribing antibiotics. This was achieved by linking these constructs to appropriate behaviour change techniques. The basis for our choice of target constructs is strengthened by the established predictive utility of the theoretical models we used in this process. Likewise, the behaviour change techniques used are also supported by a substantial evidence-base for their effectiveness across a range of settings [33,34]. Thus the final interventions are underpinned by a robust scientific rationale with which to explain "why and how" we expect each intervention to have their effect, and are placed within a sound theoretical framework that guides a process for their evaluation and refinement.

In general, the poor reporting of intervention detail, prevents replication. Such inadequate description of implementation interventions hinders the development of a cumulative science of implementation. We have tried to illustrate here the type of description of intervention components that will make it possible to replicate their essential features. By describing the interventions in terms of discrete and identifiable behaviour change techniques we are clearly differentiating between the key components of the intervention content (the proposed "active ingredients") and the method by which the intervention was delivered (i.e. as a paper-based task). Such differentiation makes it possible to investigate whether the same behaviour change techniques differ in effectiveness across other modes of delivery, whilst also offering the potential to explain differences in effectiveness across different settings. Routine reporting of detailed description – such as we provide here – would greatly enhance the replicability of implementation studies

The systematic approach used in this study was constrained in two ways. Firstly, the choice of target constructs was limited to those which predicted both simulated and actual prescribing behaviour. We applied this limitation because an evaluation of these interventions will be generalisable to the real clinical context only if there is close correspondence between the measures of intention, simulated behaviour and actual behaviour. However, external validation for our choice of target constructs is provided by Walker et al 2001, as our target constructs are represented in the discriminant beliefs identified by these authors [17]. Secondly, the chosen mode of delivery (paper-based and postal survey) influenced both the choice of behaviour change technique and the construction of the intervention components. A secondary aim of this theory-based approach is to develop methods for "pre-testing" and optimising the potential effect of interventions (implementation modelling experiments) prior to their use at service-level. Hence, a final consideration was the feasibility of using the techniques in both a modelling experiment context and a service-level randomised controlled trial. Our choice of behaviour change techniques was thus further influenced by their adaptability to the real-world setting.

## Conclusion

We have demonstrated that it is feasible to develop interventions to change professional practice that are underpinned by a robust, scientific rationale. Theoretical models, empirical data and evidence-based behaviour change techniques were integrated systematically to produce two interventions that aim to change clinical behaviour. This approach is a way forward towards creating a scientific evidence-base relating to the choice, development and delivery of effective interventions to increase evidence-based clinical practice.

## Competing interests

The author(s) declare that they have no competing interests.

## Authors' contributions

All authors contributed to the conception and design of the study and approved the submitted draft.

## Pre-publication history

The pre-publication history for this paper can be accessed here:



## Supplementary Material

Additional file 1 The graded task intervention. A copy of the paper-based graded task intervention as presented to participants. Click here for file

Additional file 2 The persuasive communication intervention. A copy of the paper-based persuasive communication intervention as presented to participants. Click here for file
